# The needs, barriers, and opportunities perceived by health professionals for an online competency-based interprofessional course to enhance the care of older adults with chronic musculoskeletal pain

**DOI:** 10.1080/10872981.2023.2172755

**Published:** 2023-02-06

**Authors:** Marta Martín-Carbonell, Yuli Suárez Colorado, Doriam Camacho-Rodríguez, Maria Yaquelin Expósito-Concepción, Salín Touchie-Meza, Doris Sequeira Daza, Luz Karinne González Julio

**Affiliations:** a Psychology Department, Universidad Cooperativa de Colombia, Santa Marta, Colombia; b Nursing Department, Universidad Cooperativa de Colombia, Santa Marta, Colombia; c Nursing Departament, Universidad del Norte, Barranquilla, Colombia; d Medicine Department, Universidad Cooperativa de Colombia, Santa Marta, Colombia; e Nursing Department, Health School, Universidad Central de Chile, Valparaiso, Chile

**Keywords:** Older adults, chronic musculoskeletal pain, Colombian healthcare professionals, pain education, qualitative research

## Abstract

**Background:**

It is recommended that continued education in pain should be supported using information and communication technologies (ICTs), but there are gaps about the previous competencies of health professionals, especially in low- and middle-income countries. This study characterized the competencies of professionals in the Colombian Caribbean, the circumstances that favor and hinder the development of appropriate care, as well as the preferences, expectations and attitudes toward an educational intervention supported by ICTs.

**Methods:**

We used a qualitative, phenomenological approach, combining documentary review and data obtained through observation, an online survey and two focus groups involving 55 healthcare professionals (physicians, nurses and psychologists) of varying experience. For the analysis and integration of results, the Capacity, Opportunity, Motivation and Behavior model and the Theoretical Domains Framework were used as references. The Consolidated Criteria for Reporting Qualitative Research (COREQ) checklist guided the reporting of this study.

**Results:**

Competency deficits were found regardless of the profession and experience, which are related to inadequacies in undergraduate and post-graduate training. Structural problems in Colombian society and healthcare service organization were also discovered, which were considered unmodifiable barriers and have been aggravated by the social, economic and health effects of the pandemic. The main modifiable barriers found were as follows: pain underestimation and under-treatment of older adults, as well as stereotypes regarding old age and pain, lack of knowledge of the psychosocial determinants of pain and of techniques for its assessment and treatment, overestimation of pharmacological treatment and failure to update pharmacological and non-pharmacological techniques.

**Conclusions:**

Recommendations for the design of the educational intervention were established as follows: favoring non-synchronous resources, facilitating synchronous activities of short duration, facilitating permanent access to information and resources and generating incentives for continuing education, such as certification, institutional recognition and encouraging popular recognition.

## Background

Pain is the most frequently reported symptom by Colombians over 5 years age in outpatient and emergency consultations [1]. According to the Colombian Association for the Study of Pain (ACED by its acronym in Spanish), degenerative osteoarthrosis is the most common cause of osteoarticular pain in older adults, affecting more than 80% of them and causing pain, discomfort and varying degrees of disability [2]. Moreover, the National Study on Health, Well-being and Aging found that pain negatively affects functional independence and the perception of the quality of life of older adults and their families [3]. Sin embargo, pain in elderly adults, especially musculoskeletal pain, is often underestimated and undertreated. Data on medication consumption in Colombia suggest that pain is treated exclusively with analgesics in approximately 57% of cases [1]

Citing information on the Fact Sheet 2 shared by the International Association for the Study of Pain (IASP): ‘*Despite extensively developed and freely available curriculum resources*, *adoption of pain content into entry-to-practice programs is agonizingly slow*. *Until now*, *most health professionals have learned pain management only through an* “*informal curriculum*” *in clinical settings that perpetuate a culture of stigma and inadequate pain care practices*’ [4]. Recently, Ampiah et al., [5] found insufficient information on the capacity of healthcare professionals to deliver interventions in low- and middle-income countries and weaknesses in their training, leading to inadequate attitudes and practices.

In relation to training in pain management in Colombia, a study by León et al., (2007) revealed serious shortcomings. This situation does not seem to have improved, as (Gónima (2019) said:

Research carried out indicated the following three facts:

1. that general practitioners who graduated in Colombia before 2007 received deficient training in the diagnosis and treatment of pain, that in 2016, that situation had changed little and that in this time and near future, no substantial changes are in sight;
2. that the current and future supply of physicians in all clinical specialties is insufficient;
3. that the deficit of specialists in the area of pain medicine and palliative care is more notorious and there is an expectation of an increase in that demand in the next decade (Gónima, 2019, p.73).

This situation is not exclusive to medical physicians or to Colombia. Similarly, there is evidence of a low level of undergraduate and post-graduate training in pain among nursing staff [6] Other professions that must participate in the basic care of older adults with pain, such as psychology, receive even less training and psychological intervention is often considered dispensable and secondary [7].

The IASP recommends that continued education in pain should be supported using information and communication technologies (ICTs) (Watt-Watson & Hogans, 2018). Similarly, ideas about the potential usefulness and impact of ICTs are welcome in Colombia [8], but unfortunately, we found only two offers of online training in pain, specifically aimed at nursing professionals [9]

Considering the aforementioned information gaps, we are working the project *‘Design and pilot study of an online course to empower health professionals in the care of older adults with pain in Santa Marta, Colombia’*. The IASP Consensus Document on Core Competencies in Pain Management, the IASP Recommendations for Pain Management [10] and the IASP Recommendations for Pain Management in Older Adults [11] serve as a guide to establish the core competencies that should be included.

The first step of our project was to identify who is providing care and when and how care is being provided to older adults with pain, particularly musculoskeletal pain, in the city of Santa Marta; to characterize their level of development of the basic competencies recommended by the IASP – and the circumstances of daily practice that favor and hinder the development of appropriate pain care for older adults – and to identify the preferences, expectations and attitudes of health professionals toward an educational intervention supported by ICT.

This report shares the results and reflections derived from the activities we conduct to achieve these objectives.

## Methods

### Methodological framework

We chose a qualitative approach as this type of research aims to understand situations without neglecting the ‘subjective’ perspective of the researcher, who is assumed to be both the subject and object of the study [12].

We worked from a phenomenological perspective as our aim was to describe and understand the phenomena from the viewpoint of health professionals through the analysis of specific discourses and themes and to search for their possible meanings [13]. At certain points in the study, we used quantitative analysis strategies (percentage and frequency distributions) to support the identification of patterns of meaning in the data, as recommended by various authors [14,15]

For the collection and primary analysis of information, we considered the recommendations of Sidani [16]. We combined the empirical approach (literature review with a narrative and unstructured approach) and the experiential approach (which included a survey of focus groups of healthcare professionals and the criteria and experiences of the experts who were part of the research team). To increase the validity of the findings, we used a triangulation of informants technique.

Analytical induction was used for the analysis and integration of the qualitative data. To integrate the results, in addition to the strategies recommended by Sidani, the contributions of the COM-B models (Capacity, Opportunity, Motivation and Behaviour) were taken as references according to Cane et al [17]., and the Theoretical Domains Framework (TDF) [18]. Both models have been cited in many peer-reviewed publications and have been successfully used for designing behavioral change interventions in healthcare professionals [18] and in the general population [19]. Several intervention studies have also used these models to design good practices in musculoskeletal pain care [20–22]

### Aspects considered in designing the strategies for data collection

In the process of data collection, it was necessary to consider the following decision points:

1. To determine when and how older adults with pain, especially musculoskeletal pain, are cared for, as well as who provides care in the city of Santa Marta.
2. To be aware of and understand the level of severity and importance given by healthcare professionals to musculoskeletal pain in older adults.
3. To be aware of and understand the perspective of healthcare professionals on the barriers and facilitators of daily practice in the care of older adults with pain.
4. To identify and characterize the weaknesses and strengths in core pain care competencies in older adults.Because our aim is to propose an educational intervention, the following point is also relevant:
5. To identify customs and preferences of healthcare professionals in Santa Marta regarding the structuring, organization and accreditation of post-graduate education, as well as their disposition toward an ICT-based educational intervention.Note that the project was conceived in 2019 before we could foresee the major changes in everyone’s lives that would occur just a few months later due to the pandemic; therefore, we had to include the following aspect:
6. To identify potential changes due to confinement and the rise of remote working, as well as the pressures and risks that healthcare professionals face.

Themes and specific strategies were defined iteratively as the identification and characterization progressed, based on the literature review and the information obtained from the data collection, and new themes and strategies were defined until the information was saturated.

## Sampling strategies and participants

The ‘case’ being studied is of healthcare professionals in the Colombian Caribbean, given the proximity of the cities along the Caribbean Coast (Santa Marta, Barranquilla and Cartagena). These cities share an identity that minimizes the cultural pattern heterogeneity. Another common feature in Caribbean cities is a shortage of healthcare professionals with specific training in pain care, as well as a scarcity of post-graduate training programs, a situation that affects practically the whole country.

Including multiple perspectives was essential and that it helped determine the composition of the research team and the selection of informants. Therefore, purposive diversity sampling was used to identify various viewpoints based on the following variables:

- Diversity of professions: physicians, nurses, physiotherapists, psychologists and social workers
- Different professional experiences: final year trainees, recent graduates and healthcare professionals with professional experience ranging from less than 1 year to more than 0 years
- Different experience in caring for patients with pain and older adults: from less than 1 year to more than 0 years
- Different levels of post-graduate academic training: from none to post-doctorate
- Different departments of the Caribbean Region: Magdalena (Santa Marta), Atlántico (Barranquilla) and Bolívar (Cartagena)
- Socio-demographic differences: Gender and age.

According to their role in the research, participants were classified as follows:

1. Research team members: considered experts not only because of their level of training in the subject, but also because they are leaders in training the direct beneficiaries of the educational intervention (physicians, nurses and psychologists from Santa Marta) and are professionals directly linked to the clinic.
2. ‘Doorkeeper’: people who helped us recruit informants such as directors of universities and health institutions
3. Informants: senior students and professionals from various health disciplines, with various levels of professional experience linked to care for the elderly.

The study included 55 healthcare professional informants who participated in at least a activity developed to gather information.

## Strategies for recruiting informants

All activities were conducted virtually due to mobility restrictions imposed for pandemic control and biosecurity measures.

### Instruments

- **Online survey**

The survey was designed by two psychologists from the research team. The wording of the questions, the order and format of the presentation and contents of the questions were evaluated by the other researchers in the team (i.e., a physician, three nurses and another psychologist) who proposed corrections until a consensus was reached. The survey contained closed-ended questions with multiple choices and open-ended questions. It was an anonymous survey, and subjects were guaranteed anonymity at the time of requesting informed consent. The survey consisted of two sections. Section 1 contains an explanation of the study and a request for informed consent. If the person expressed their agreement, they moved on to the next section. Section 2 had questions to obtain socio-demographic and professional information. These questions aimed at obtaining information about the institutional contexts in which they work and identifying professionals who care for older adults with pain, the frequency and importance of pain care for older adults, healthcare facilitators and barriers and criteria and preferences about the possibility of receiving training on the subject.

The survey was conducted online using a Google form. It was shared using social media and email. Furthermore, the ‘snowball’ strategy was used. Thirty-four healthcare professionals from various Colombian Caribbean regions responded to the survey: 36.4% from the two main cities of the Magdalena Department, 27.3% from the Department of Atlántico and 36.4% from other departments. A total of 41.2% were nursing professionals, 29.4% were physicians, 26.5% were psychologists and 2.9% were other professionals, such as physiotherapists, social workers and administrative staff working directly with patients. Of the participants, 34.7% were men and 65.6% were women, with a mean age of 6 years (standard deviation = 12.65). Also, 45.5% reported post-graduate training. Time of professional experience (mean = 21 months, standard deviation = 119.60).

- **Focus groups**

The participants were invited to the focus group by the researchers who had professionally collaborated with them. A nurse with experience in this type of technique led two focus groups with the help of a psychologist and another nurse, as observers.

- Focus Group 1: experienced healthcare professionals working in various health institutions in Santa Marta who provide care for older adults with chronic pain problems. Seven people participated as informants, 50% of whom were women. The participants included two physicians, three nurses and a physiotherapist, all with experience in health care and two others with experience in university teaching. The professionals were in middle adulthood and only one was in older adulthood.
- Focus Group 2: students in their final semester or those who recently graduated from healthcare-related courses at the University of Barranquilla. Six people between 19 and 3 years of age participated. The participants were women, with two nursing students, one nursing graduate, one psychology graduate and two psychology students.

Group meetings were conducted using the Microsoft Teams platform and were recorded with the consent of the participants. Each meeting lasted approximately one and a half hours. Both meetings were based on a ‘script’ with guiding questions focused on the objectives.

## Strategies for analysis and integration of results

The analysis adopted an inductive approach. It was done in two steps: first, analysis by techniques and stages; second, integration.

The following activities were carried out in stage 1: 1) identification of the requirements that healthcare professionals must meet to be selected as caretakers and initial informants, definition of strategies for recruiting informants and development of instruments for obtaining information and strategies for their application; 2) design and implementation of an online survey and a focus group of a previous task; 3) analysis of the data obtained from each technique; and 4) integrative analysis of the data obtained.

The following activities were carried out in stage 2: 1) because of stage 1, we decided to conduct a second focus group, including people with different education levels and experiences and from another region of the Caribbean, to verify hypotheses and refine the general themes and sub-themes; 2) analysis of the results of the second focus group; and 3) final integration.

For the analysis of the data obtained in the survey, quantitative analysis (percentage distributions) was combined with content analysis of the open-ended responses, which was carried out by a psychologist with experience in this type of analysis, considering the recommendations of various authors to consider three moments (discovery, coding and relativisation) (Anguera et al., 2020). The analysis was discussed and finalized by the team.

The information obtained in the focus groups was analyzed using ATLAS.ti software (ATLAS. ti, n.d.) by two psychologist researchers who, after familiarizing themselves with the discourses, used open coding to generate initial codes and categories. The codes were discussed in the context of scrutinizing deviant cases, checking for confirmatory or challenging evidence within the dataset and interpreting patterns. At all stages of the analysis, we considered a construct to be relevant if it was (a) frequent, (b) participants’ attitudes and beliefs about the construct were contradictory and/or (c) the construct was associated with strong attitudes and beliefs [18].

We met as a team via regular tele-conferences to discuss, compare, synthesize and map relationships between findings; compare our findings with our theoretical framework; and generate interpretative insights about the data [23]. We discussed the ongoing results via online meetings and/or individual phone calls with the rest of the research team members. The integrative analysis was initially carried out by the first author of the article and subjected to discussion and improvement by the team. For this integrative analysis, as mentioned above, we relied on the combination of the COM-B model according to Cane et al [17]. and the TDF [17,18,24]. The COM-B model helps understand how the capacities of individuals to modify their behavior, opportunity (understood as environmental factors that influence individual behaviours) and motivation (understood as the willingness to change) can be used to generate actions that have a positive impact. The TDF [17–24] was used as a ‘theoretical lens’ to analyse cognitive, affective, social and environmental influences on behavior.

## Results

Table 1 shows the socio-demographic and professional information of the participants.

**Table 1.** Socio-demographic and professional information of the participants.

Variable	Category	Frequency	Percentage
Gender	Female	39	70.9%
	Male	20	29.9%
Age	0 years	23	40.7%
	1 years	33	59.2%
	No information	3	3.6%
Health Area	Psychology	13	21.8%
	Medicine	15	23.6%
	Nursing	22	38.1%
	Other	3	5.4%
	Student nurse	3	3.6%
	Psychology student	3	5.4%
Pain experience	Yes	40	72.7%
	No	19	27.2%
Participants	Survey	39	61.8%
	Focus Group 1	7	10.9%
	Focus Group 2	6	14.5%
	Research team	7	12.7%

Table 2 summarizes the relationship between the COM-B components and the TDF domains, along with the categories and codes derived from the integration of the results.

**Table 2.** Integration of the results: COM-B components, TDF domains, categories, and codes.

COM-B component	Constructs	Emerging categories	Codes
**Capacity to change**: Knowledge	Environment awareness	Who cares for older adults in pain	Professionals, professional experience
		Where and when to care for older adults in pain	Institutions, services Care pathways
		Frequency and importance given by professionals to musculoskeletal pain in older adults	Frequency of the motive for consultation Assessment of the importance given to musculoskeletal pain
	Technical and procedural Knowledges	Core competencies	Emotions, Depression, and pain Family abandonment Support Assessment (visual analogue scales, laboratory diagnostic tests, X-rays, MRI) and management (pharmacological, alternative medicine, support, relaxation, multidisciplinary). Contextual factors (manifestation particularities associated with ageing, risks of polypharmacy, aetiology-related, service-related and level of care), attitudes toward aging
		Academic background	Specific training in undergraduate, postgraduate, continuing education
		Topics of interest for post-graduate training	Non-pharmacological techniques and alternative medicines Mental health and pain Specific professional interests unrelated to chronic musculoskeletal pain Importance of educating family members
**Motivation to change** Physical environment		Impact of the pandemic on health services	Predominance of tele-consultations Decrease in services Decrease in demand from older adults
Social influences	Remote work	Rejection of virtual meetings	Difficulty in convening meetings Low attendance Not sharing camara Few responses to online survey
		Connectivity and power failures	Disconnection during meetings Not sharing a camera
	Multidisciplinary	Recognition of multidisciplinary work	Non-pharmacological therapies, alternative medicine, mental health screening, physiotherapy, social work, multidisciplinary work claim, mental health
	Commitment	With the team	Attendance and willingness to co-operate
		With their profession and institutions of belonging	Defending their institution Positive feedback from health professionals
**Opportunity to change**: Context	NHS	Weaknesses and failures of the NHS	Limitations to include studies and treatments, short consultations (5 min), inadequate physical spaces for older adults, poor remuneration, lack of resources and supplies to work with
	Resources for online training	Devices and connectivity Use of platforms for communication	Smartphones, tablets, laptops Internet connection Experience with Teams, Meet, Zoom
Capacity beliefs	Perceived competence	Recognition of competence deficits	Acknowledging that did not have undergraduate and post-graduate training Identification of topics in which training is needed Need for post-graduate certification Limited concept of emotional support linked to stereotypes about old age Low Importance given to training needs
Empowerment	Constraints to performance	Inability to change the environment	Limitations of the HPE Connectivity problems Frequent power failures
Emotions	Emotions	Negative	Frustration, hopelessness
Intentions	Professional development	Longing for a high-quality practice	Criticism of malpractice Criticism of the limitations imposed by HPSs Expressing the sensitivity of older adults for to alleviation of suffering
		Specific pain training	Wishes to receive
		Specific to online training	Accept training Available time requirements

Described below what we learned from this analysis on the core competencies of ‘frontline’ healthcare professionals. Additionally, their preferences and training needs, as well as the aspects that can be conceptualized as barriers or opportunities for a change in the behavior of health professionals to improve their competencies, are discussed.

## Capacities (the starting point …)

### Who cares for older people in pain, where and when

The information on the human resources involved in healthcarein Colombia is limited. According to Aura et al., (2014) in 2010, there were around 226,600 professionals in the health sector in Colombia. Of this total, 77,000 are physicians, which gives a ratio of 1.7 physicians per 1,000 inhabitants. Next in numerical importance are nurses (42,000), therapists (40,000), and dentists (40,000). There is no statistical information to infer the number of health professionals who care for the elderly population at the different levels of care.

Our study allowed us to identify what types of health professionals, in the Colombian Caribbean, frequently care for the elderly, and therefore, can detect or receive requests for pain care. They are General practitioners, practitioners of alternative and/or traditional medicine (not necessarily with academic or professional training), nurses, clinical psychologists and social workers linked to multidisciplinary teams in clinics or hospitals

Not all of these professional workers provide care to older adults in pain at the same frequency. As expected, the most frequent professionals caring for older adults in pain are physicians (as recognized by 91,2% of participants) Among non‑medical professionals, those who most frequently see older adults in pain are nurses (mentioned by 85,3%) and physiotherapists (identified by 73%), but fewer psychologists (mentioned by 61,8%) and social workers (only mentioned by 23,5%). For example, medical participant AZ from the focus group 1 said, *‘I imagine that the patient can be routed to consult with the psychologist, but it is not easy’*.

The locations in which these professionals come into contact with older adults who may need pain care are varied, with some working at the same time in different types of institutions.The most frequent services where older adults with pain are observed (although this may not have been their main reason for consultation) are outpatient clinics, nursing homes and rehabilitation centres. Less frequently, cases may occur in the emergency department. However, it has been noticed that a large proportion of older adults seek treatment in alternative medicine centres.

**Figure 1.**
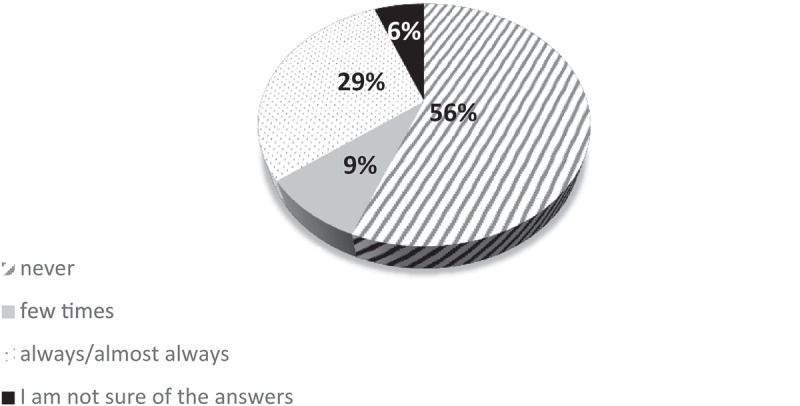
Frequency with which older adults request care for pain in non-specialized consultations.

In the survey and focus groups, participants agreed that the musculoskeletal pain was a problem that occurred frequently in older adults (Figure 1), although it was not the main reason for consultation at many occasions, as this depended on the type of service. For example, participant Doctor VC in the focus group 1 said ‘*There are many patients who come for consultations and their main consultation is not about pain. It is like an ancillary symptom that the patient comments on, although some also directly go seek for pain relief’*.

**Figure 2.**
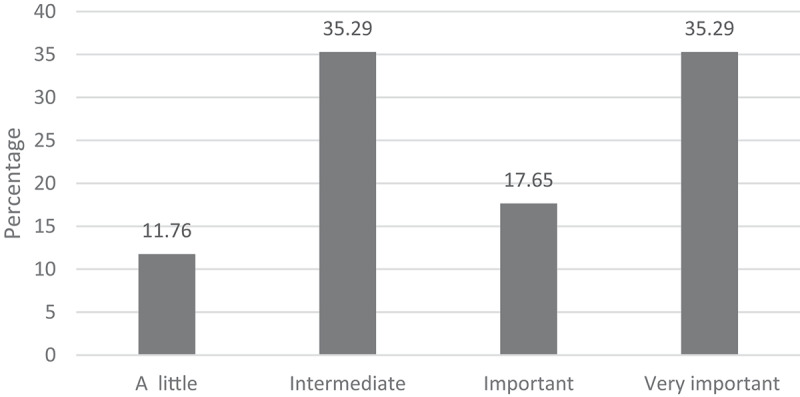
Importance given by health professionals to pain in older adults.

However, healthcare professionals do not always give importance to the complaints of older adults (Figure 2).

Care routes for the elderly with pain are not defined. The information obtained through the different means showed that professionals are unclear about the procedure to followed when older adults present with pain. It was also found that there may be a disconnection between the different levels of care and services as the professional may lose contact with the patient once they leave their service (Nurse PE, participant in focus group 1: *‘As a clinic, we do not have a follow-up programme’*).

### Technical and procedural knowledges

Concerning the main knowledge needed, both in the online survey and in the focus groups, the predominant opinion among the informants was that, in general, healthcare professionals have a medium – low level of knowledge in pain management, regardless of their profession.

Concerning assessment, physicianss and nurses (mainly but not exclusively those with more professional experience) stated that they were familiar with pain self-assessment scales but did not use them frequently in practice due to time constraints. Likewise, they complained about a lack of training and opportunity to conduct specialized studies (radiological, laboratory, etc.) due to limitations imposed by the HPE. The assessment of psychological and social factors relevant to the care of older adults (e.g., beliefs, attitudes and behaviours) was not mentioned

They report that medication-only pain management predominates, along with a lack of pharmacological management updates (Example: participant Doctor BT in focus group 1: ‘They only prescribe acetaminophen’, and the rest of the participants agree). They were critical of the predominance of exclusively pharmacological treatment and of the risk of polypharmacy in older adults (Physician participant AZ in focus group 1: *‘ … polymedicated patients, the risk of prescribing x or y medication for these patients is not considered … ’*).

It was found that healthcare professionals recognize, value, demand and respect the multidisciplinary approach and the potential contributions of different disciplines toward the treatment of pain in elderly adults. They emphasize the importance and contributions that should be made by psychology, physiotherapy and social work, as well as the importance of integrating nonpharmacological therapies, but complained that patient access to these alternatives is deficient (participant Nurse PE, focus group 1: *‘Sometimes they ask them to see a physiatrist. I encountered a case when an institution referred to a patient for acupuncture, but authorizing acupuncture was a difficult process’*).

They emphasized the importance of providing effective support to older adults through behaviors that express affection (Example: Participant Doctor AZ of focus group 1, while referring to the stage in which she worked in the nursing home, said *‘I hugged them, talked to them to distract them; we talked about something other than their relatives because they suffer from neglect’*); but they had stereotypes about older adults being ‘fragile, abandoned, mistreated, poor, and lacking in affection’, and in consencuence, they use paternalistic behavior. In fact, in an open-ended question in the survey on aspects to be improved in pain management for older adults, several participants spontaneously highlighted the lack of empathy from healthcare professionals as the main problem, with phrases such as ‘lack of empathy’ (six participants) and ‘they do not pay attention to it’ (eight participants). These shortcomings are primarily due to problems with Colombia’s healthcare organization, as seen below.

**Figure 3.**
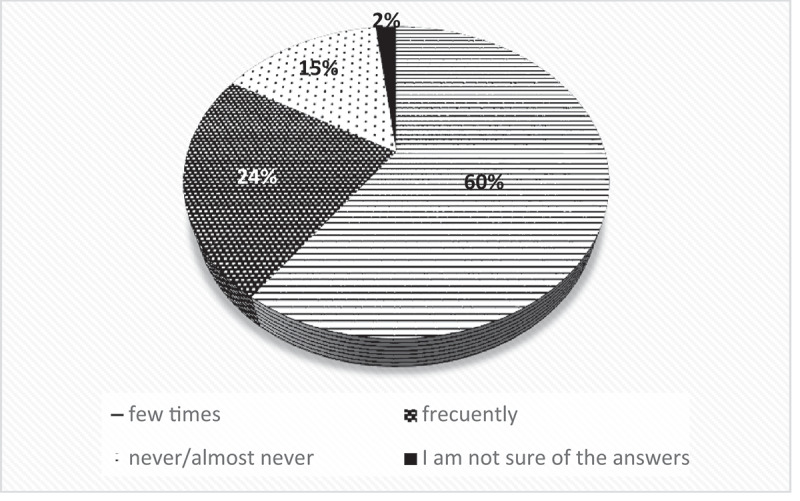
Evaluation of the frequency that healthcare professionals show empathy for the pain experienced by older adults.

Professionals (in general, regardless of profession or time of experience) could recognize and spontaneously express some of the emotional aspects related to the experience of pain (Medical participant AZ of focus group 1, who has 0 years of experience, said, ‘*I had to manage many cases of musculoskeletal pain associated with depression’*, and participant Psychologist MC of focus group 2, who recently graduated, said, *‘It is necessary to provide emotional support, not only the management of physical pain’*). In contrast, informants were predominantly of the opinion that most of their colleagues do not provide adequate emotional support or show empathy toward older adults in pain (Figure 3).

The professionals interviewed attributed their ability and interest in providing emotional support to their personal experiences and life history. (Medical participant AZ from focus group 1: ‘*Being affectionate is paramount for me, even before prescribing medication’. Personally, I saw him as if he was my grandfather … one associates it with helping these older adults’*). Despite emphasizing the importance of emotional support, it was evident in the focus groups that they were unable to verbalize concrete psychological strategies and techniques beyond ‘support’, including those who were psychology students and graduates who were interviewed.

Physicians and nurses recognized that aging could establish particularities in the manifestation and course of pain (Participant Nurse RP in focus group 1 said, *‘Pain manifests differently in older adults, and older adults respond differently to drugs’*), whereas psychologists pointed to the cognitive deterioration and emotional and family problems of older adults (Participant psychologist LH in focus group 2 said, *‘If they are depressed, their hospital stay will be longer, and their recovery is slower’*).

In the contextual aspects to be considered in pain management, physicians and nurses considered the differences in the approach to acute and chronic pain, the management of a critical patient and the risks associated with aging (Participant physiotherapist AR, focus group 1: ‘*From the perspective of critical care*, *not managing pain in older adult patients is one of the main causes of decompensation and it is not necessarily due to the underlying pathology … but because pain is not managed well’ and* participant Nurse PE of focus group 1: ‘*They are at risk of any adverse event precisely because of the morbidities and age of the patient*’). The psychologists focused on abandonment and family abuse (participant Psychologist MC of focus group 2: ‘*You* must consider that *they are left at home and are never visited*. *True*, *they are in pain*, *but how much* is their *loneliness affecting them?*’).

Professionals agreed that they had received no specific undergraduate or post-graduate training in pain care or in the care of older adults, apart from specific and limited content in the undergraduate courses of the different professions and some occasional talks sponsored by laboratories. (Nurse PE, focus group 1: ‘*No*, *I have not received training*’; Doctor AZ of focus group 1: ‘*Not really training or post-graduate courses*, *only invitations to seminars*’; and Nurse RP of focus group 1: ‘*I have not had any training in pain courses, diploma, or specialization courses*. *I learn from my clinical practice*’). They attribute their knowledge to practical experience, and in the case of students and recent graduates, to training practices that they had the opportunity to conduct during their undergraduate studies (Nurse DG in focus group 2: ‘*In palliative care classes*, *I remember a pain forum but not much more*. *During practice on the floor*, *you learn*, *do assessment and pharmacological treatment*’).

## Motivation, opportunities (and barriers …)

### Context and environmental resources, perceived competence, emotions and intentions

**Figure 4.**
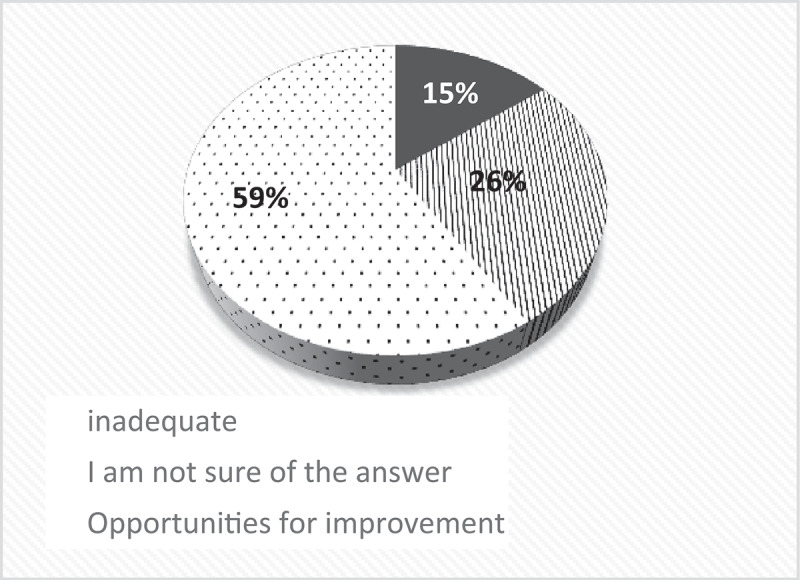
Quality of care received by older adults according to healthcare professionals.

Both in the focus groups and in the online survey, there was a predominant view that older adults with pain do not receive basic quality care, as shown in Figure 4, which they attribute mainly to deficiencies in the health system in Colombia: limitations in indicating studies and treatments, short consultation time (5 min), inadequate physical space for older adults (although this varies in the different institutions), poor remuneration and lack of resources and supplies.

The response options were: Excellent, Satisfactory, Opportunities for improvement, Insufficient, I am not sure of the answer. Note that no one answered Excellent or Satisfactory

It also became evident that the professionals feel that they cannot do anything to change this situation and have accepted it, although it causes them emotional discomfort (Nurse RP, focus group 1: ‘*physicians have many limitations for patient care as the EPS do not authorise many studies that are important*, *nor do they allow them to freely indicate the treatment …* ’).

Younger people, with less work experience, identified having a deficit in skills in pain management of older adults (Psychologist MC, focus group 2: ‘*It is necessary to include these issues in some subjects*, and nurse YB, focus group 2: ‘*A course or a diploma would be great*. *I want to have specific training on this subject, as one only knows the basics*’). Professionals with more professional experience stated that they would like more training (Doctor VC, focus group 1: ‘*It would be excellent to receive a course*. *I would be interesting to be trained because what we know is what we have learned during practice*’), and in this regard, they agree with those with less experience, but they believed that the main barriers to care are due to the failures in the health system in Colombia rather than a lack of knowledge and skills of professionals (Nurse PE, focus group 1: ‘*We are in a health system that often limits us*’. ‘*The physicians may have all the knowledge … but our health system is limiting*’).

Concerning intentions and willingness for continued education in pain, the desire for high-quality professional practice was evident. Professionals expressed their willingness to receive post-graduate training (Doctor AZ, focus group 1: ‘*all training is welcome*’), but there were differences between experienced and less experienced professionals. In the latter, there is a demand for formal and certified post-graduate academic training that allows them not only to achieve competencies but also to move up in their professional life (Nurse DG, focus group 2: ‘*I want to work with older adults, even in a nursing home*, *and I think it would be great to have specialised training in this area*’). Professionals with more experience, although expressing their willingness, were not very enthusiastic because they did not think it would have a major impact on the quality of services as explained above and/or because their interests were focused on other areas.

The number of hours per week they said they would be willing to devote to training is limited (between 2 and 4 h), even for the least experienced. In relation to the interests and preferences of topics for training, they stated the following points: updating on pharmacological pain management (although also related to the limited possibilities of prescription), the need and high motivation to learn about nonpharmacological therapies with an emphasis on mental health and its relationship with pain, education and emotional support for older adult patients with pain and their families (Doctor BT, focus group 1: ‘*Neural therapy*; *we have excellent physicians in alternative therapies*’ *and* Doctor VC, focus group 1: ‘*Very convenient to have the geriatrician*, *general practitioners*, *head nurses*, *nursing assistant*, *a physiotherapist and a physiatrist*’). Likewise, the need to educate patients and relatives emerged due to their frequent unfair attacks on healthcare professionals when they try persuading the professionals to solve problems of access to services and/or treatments that the professional cannot solve. Other issues, not directly related to musculoskeletal pain but of interest to professionals involved in the care of older adults, also emerged. As highlighted by the nurses, these issues include the need for training in palliative care and training in complex techniques for managing acute pain.

Informants have devices, such as mobile phones and tablets, but reported that frequent power and/or connectivity failures may affect online training, which, in fact, members of the research team have experienced (and suffered). One example was that in focus group 1, a nurse logged in at the beginning but could attend for only 0 min because she lost connection.

The analysis of the communicational and attitudinal aspects derived from the observation of the focus groups, as well as from the answers to open questions in the survey, revealed social influences that can be seen as opportunities, such as commitment to the profession and to their institutions of belonging. For instance, in the online survey, among the strengths were ‘*professionalism*’, ‘*human quality*’, and ‘*in my institution all medicines are available*’.

The physical environment related to the restrictions of the pandemic is an important barrier because outpatient health services have been largely restricted to tele-consultation, which hinders a correct assessment. Additionally, due to administrative indications and/or limitations of time and knowledge of the tele-consultation tools, everything apart from the main reason for the consultation is ignored. If the patient manifests with pain that was not initially observed during the consultation request, it is not managed (in most cases). Alternatively, priority has been given to pandemic-related consultations, which reduces the number of professionals and services offered for other problems, and older people do not seek care because of the fear of contagion.

Furthermore, the rise of teleworking, the requirement to be connected to communication devices (mobile phones, computers, etc.) for extended periods, and the overload of demands and offers of training, online meetings and webinars, as well as a lack of skills and knowledge in the use of ICTs, are causing a rejection of these modes of communication, which are practically the only ones available on many occasions. An example of this was the difficulties in arranging online meetings of the team members themselves and with informants. Paradoxically, this has led to health professionals becoming familiar with the use of some platforms used in virtual education, such as Teams, Zoom and Meet.

## Discussion

Firstly, our results confirm that in the Colombian Caribbean, there are quality problems in the care of older adults with pain that have been widely recognized and highlighted in other contexts (Croft et al., 2020; Guerrero et al., 2014; IASP, 2018; van der Gaag et al., 2021). These problems are related to inadequacies in undergraduate and post-graduate training of Colombian health professionals [25–27], as well as structural problems in Colombian society and in the health services themselves [28].

Concerning deficiencies in training, the document of recommendations for the transformation of medical education in Colombia recognizes that: *… ‘in terms of the set of competencies acquired by general practitioners*, *there are reports of limitations in performance*, *lack of safety in the management of patients*, *precarious knowledge of the most relevant public health issues and difficulties in communication and teamwork’* (Ministry of Education & Ministry of Health and Social Security, 2017, p.8).

In fact, Franco-Giraldo (2015) states that the fundamental problems of medical training in Colombia can be summarized as follows: lack of social commitment to the health care of the population; lack of comprehensive training from technical and humanistic perspectives; the high cost of health care reflected in a high proportion of specialized physicians versus general practitioners; poor knowledge and skills of graduates in primary healthcare management; emphasis on a biological model; centralization of learning practice in hospitals; and the absence of a focus on health promotion and disease prevention at the individual, family and community levels from the start of training.

However, Colombia, like other countries in recent decades, has adhered to a model of health service provision that prioritizes specialized curative care, focuses on the provision of services by disease groups and favors commercial interests over collective ones, especially to the detriment of access to the most vulnerable populations, such as older adults [29,30].

Although no accurate diagnosis of the situation of older adults in Colombia exists, it is estimated that only 2 out of every 10 people aged 5 years and receive a pension, and among those above 55, three million live in poverty, without any protection because they do not contribute to social security, whereas those in higher strata live in loneliness and inactivity [31]. It is estimated that less than 30% of people in their 60s receive paid work of any kind (Ministerio de Salud y Protección Social, 2015) Several studies have reported that health care for older adults in Colombia is deficient; this results in a predominant dissatisfaction with the healthcare services [32–34]

Therefore, inadequacies in undergraduate and post-graduate training, socio-economic problems, and failures in the organization and administration of health services are barriers that cannot be overcome in the short-term, either by the research team or by health professionals and have implications for the design of the educational intervention. These barriers to professional performance and their impact on the quality of care are not exclusive to pain management, nor to the Caribbean Coast, as they have been pointed out by various authors, e.g., in Dentistry, by Suárez et al., (2014), in Gastroenterology, by Uribe Pérez et al., (2019) or in mental health, by Bartels et al., (2021).

However, the theoretical frameworks provided by the COM-B and TDF models allowed us to identify problems that could be improved, which confirm the need and relevance of educational interventions to promote basic competencies in pain management for older adults while highlighting ‘removable’ barriers and opportunities to be considered for their implementation and establishing realistic expectations of their short-term impact on the quality of services.

Therefore, it was possible to establish general guidelines for the design of the educational intervention, such as favoring non-synchronous resources; favoring synchronous activities of short duration; facilitating permanent access to information and resources and generating incentives for continuing education, such as certification and institutional recognition; and encouraging popular recognition with the support of the media and social networks to increase motivation and commitment.

## Limitations

This study has some limitations. When we started this project in the second half of the 2019, we had no idea about the epidemiological, economic and social situations that COVID-19 pandemic would cause, let alone its devastating impact on health professionals and older adults. Therefore, a problem that has historically been undervalued and undertreated, such as musculoskeletal pain in older adults, has lost even more relevance in the minds of civil servants, professionals and the population itself, with perhaps the exception of older adults who suffer without the possibility of accessing adequate care. We believe that this, with the fatigue associated with the excessive and almost exclusive use of ICT-based communication, made it difficult to collaborate with health professionals and managers of institutions, which forced us to resort to interpersonal and professional relationships as the main means of recruiting informants.

This may have led to a possible limitation that the responses of participants have been influenced by social desirability bias and confirmation bias, which we were unable to verify. In fact, we tried doing another focus group with participants we did not know personally, but we did not succeed in assembling them. Similarly, the online survey, although shared via social media, was responded to only by those to whom we sent the link directly and asked for their support in responding.

Another limitation of this study is that the topic guide for the focus groups was not originally established from the COM-B and TDF models, which might have resulted in some domains not being fully explored. Moreover, the use of TDF and COM-B for integrating the results was challenging due to the perceived ‘overlap’ between the domains and difficulties in assigning constructs to specific domains, as other authors have mentioned [35,36].

Finally, this study would have benefited from the perspective of older adults in pain themselves and their relatives, which was impossible due to communication and mobility limitations imposed by the pandemic.

## Conclusions

Healthcare professionals in the Colombian Caribbean identify quality problems in the care of older adults with pain that are related to inadequacies in undergraduate and post-graduate training, as well as structural problems in Colombian society and problems in the organization of health services, which are considered unmodifiable barriers. They agreed in identified deficiencies in basic competencies for the care of older adults in pain, regardless of profession and professional experience, which were aggravated by the social, economic and health effects of the pandemic. Modifiable barriers and opportunities were also identified for implementing an educational intervention to develop core competencies and to establish realistic expectations of their short-term impact on the quality of services. The main modifiable barriers identified were underestimation and under-treatment of pain in older adults, stereotypes about old age and pain, lack of knowledge of the psychosocial determinants of pain and of techniques for its assessment and treatment, overestimation of pharmacological treatment, and failure to update pharmacological and nonpharmacological techniques. Although the professionals attribute these deficiencies primarily to the problems of the healthcare system, they also recognize the need for training and are willing to receive it, they accept online training, demonstrate commitment and high motivation toward training in non-pharmacological therapies, mental health and its relationship with pain, education and emotional support for older adult patients with pain and their families, although with limitations in the time they would be willing to invest [25,26,5,37–45].

## List of abbreviations

ACED: Colombian Association for the Study of Pain
IASP: International Association for the Study of Pain
ICTs: Information and communication technologies
COM-B: Capacity, Opportunity, Motivation and Behavior
TDF: Theoretical Domain Framework
